# 172. Inpatient Antibiotic Prescribing Patterns Using the WHO Access Watch and Reserve (AWaRe) Classification in Okinawa, Japan: A Point Prevalence Survey

**DOI:** 10.1093/ofid/ofab466.374

**Published:** 2021-12-04

**Authors:** Payal K Patel, Naoyuki Satoh, Masashi Narita, Yoshiaki Cho, Yusuke Oshiro, Tomoharu Suzuki, Karen E Fowler, M Todd Greene, Yasuharu Tokuda, Keith S Kaye

**Affiliations:** 1 University of Michigan and the Ann Arbor VA Healthcare System, Ann Arbor, MI; 2 Heartlife Hospital, Okinawa, Okinawa, Japan; 3 Okinawa Chubu Hospital, okinawa, Okinawa, Japan; 4 Okinawa Prefectural Nanbu Medical Center and Children’s Medical Center, Okinawa, Okinawa, Japan; 5 Nakagami Hospital, Okinawa, Okinawa, Japan; 6 Urasoe General Hospital, Okinawa, Okinawa, Japan; 7 Muribushi Project for Teaching Hospitals, Urasoe, Okinawa, Japan; 8 University of Michigan Medical School, Ann Arbor, MI

## Abstract

**Background:**

Few studies have been done on inpatient antibiotic use in Japan and antibiotic stewardship programs with dedicated full-time equivalents are rare. We sought to better understand inpatient antibiotic use in Okinawa, Japan. We applied the World Health Organization (WHO) Access, Watch and Reserve (AWaRe) Classification to compare our findings to international literature. Access antibiotics are common front-line antibiotics, Watch antibiotics are high-priority antibiotics with toxicity or resistance concerns, and Reserve antibiotics are last-line treatments for multi-drug resistant infections.

**Methods:**

A point prevalence study was conducted in five hospitals in Okinawa, Japan on Oct 1, 2020. Physicians conducted chart reviews of all patients receiving intravenous antibiotics. Type of antibiotic, reason for use, duration, and microbiologic data was collected. The primary aim was to evaluate the proportion of patients who received antibiotics on the assessment date; secondary aims were to categorize antibiotics according to indication, class and AWaRe classification. Descriptive statistics were used to derive the distribution of AWaRe Classifications and drug class.

**Results:**

1,728 unique patients were included and 504 (29%) received ≥1 antibiotic on the assessment date. A total of 559 antibiotics were used for 504 patients and 22.0% (n=123) were for prophylaxis. Of those receiving antibiotics for treatment (N=436), 385 (88.3%) patients had a documented infection source. The most common indications for antibiotic use were pneumonia (24.2% n=93), urinary tract infection (19.7% n=76), and intraabdominal (17.9% n=69). Overall, 43.1% (n=241) of the antibiotics were categorized Access and 54.4% (n=304) Watch [Figure 1]. Cephalosporins were the most common antibiotic class (56% n=313), followed by β-lactam inhibitors (18% n=106) and narrow penicillins (8.2% n=46) [Figure 2].

**Conclusion:**

29% of inpatients in these 5 Okinawan hospitals were prescribed an antibiotic on the survey date. A majority of antibiotics used fall under the WHO AWaRe Watch classification which are antibiotics that may be more likely to cause resistance. Understanding appropriateness of antibiotics used in this population could inform antibiotic stewardship strategies and reduce antibiotic resistance.

Figure 1. Antibiotic Distribution According to World Health Organization (WHO) Access, Watch and Reserve (AWaRe) Classification

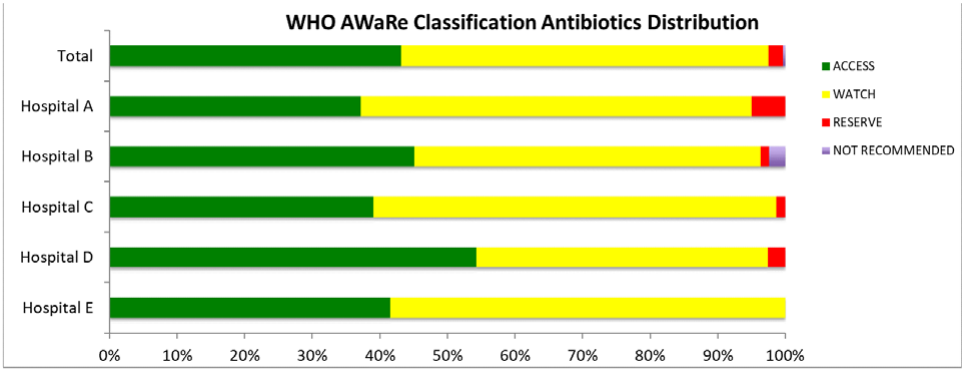

Figure 2. Antibiotic Distribution by Class in Okinawan Hospitals

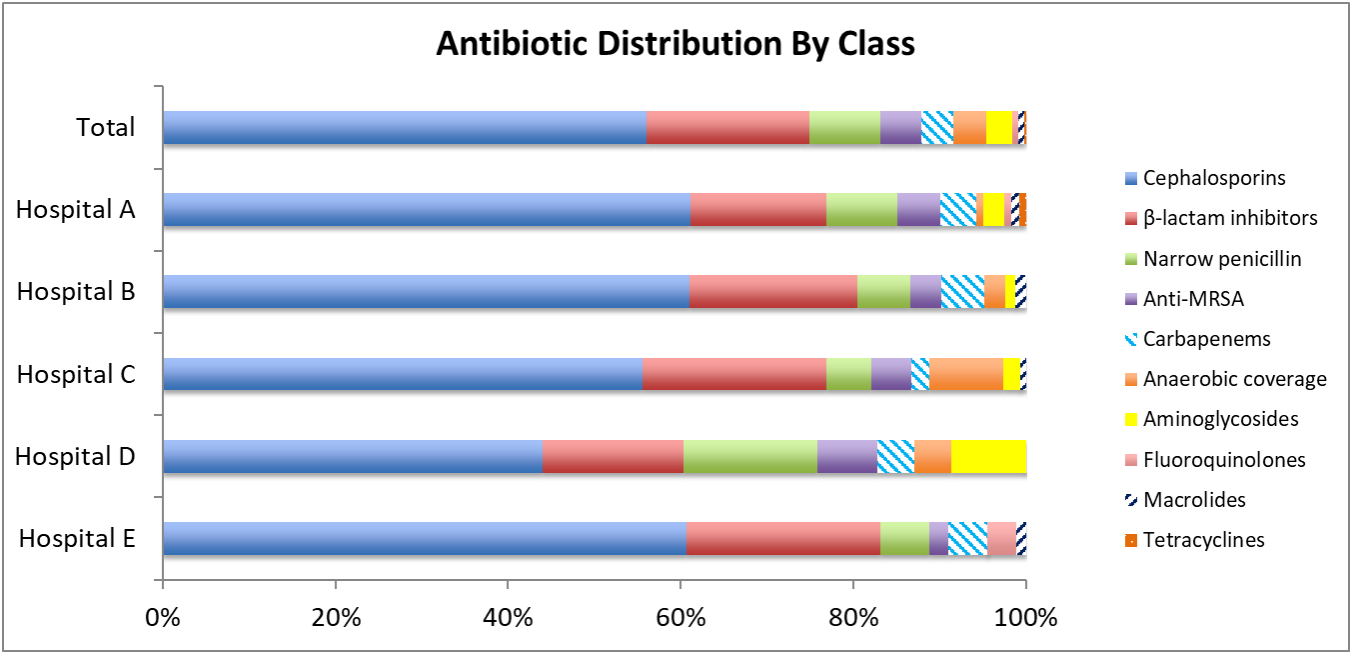

**Disclosures:**

**All Authors**: No reported disclosures

